# Sustained Therapeutic Benefits Using Image‐Guided Programming at Activation of Deep Brain Stimulation for Parkinson's Disease

**DOI:** 10.1002/mdc3.70154

**Published:** 2025-06-05

**Authors:** Jason L. Aldred, Theresa Zesiewicz, Michael S. Okun, Adolfo Ramirez‐Zamora, Okeanis E. Vaou, Leo Verhagen Metman, Corneliu C. Luca, Ritesh Ramdhani, Jennifer Durphy, Yarema B. Bezchlibnyk, Jonathan D. Carlson, Kelly D. Foote, Sepher B. Sani, Alexander M. Papanastassiou, Jonathan R. Jagid, David B. Weintraub, Julie Pilitsis, Andres Hurtado, Rajat S. Shivacharan, Benjamin Reese, Edward Goldberg

**Affiliations:** ^1^ Selkirk Neurology Spokane Washington USA; ^2^ Department of Neurology and Neurosurgery University of South Florida Tampa Florida USA; ^3^ Norman Fixel Institute for Neurological Diseases, Department of Neurology University of Florida Gainesville Florida USA; ^4^ Department of Neurology and Neurosurgery University of Texas San Antonio San Antonio Texas USA; ^5^ Department of Neurology Northwestern University School of Medicine Chicago Illinois USA; ^6^ Department of Neurology and Neurosurgery University of Miami School of Medicine Miami Florida USA; ^7^ Department of Neurology Zucker School of Medicine at Hofstra/Northwell Health Hempstead New York USA; ^8^ Department of Neurology and Neurosurgery Albany Medical Center Albany New York USA; ^9^ Inland Neurosurgery and Spine Spokane Washington USA; ^10^ Department of Neurosurgery Rush University Medical Center Chicago Illinois USA; ^11^ Department of Neurosciences Nassau University Medical Center East Meadow New York USA; ^12^ Department of Neurosurgery University of Arizona College of Medicine Tucson Arizona USA; ^13^ Boston Scientific Neuromodulation Valencia California USA

**Keywords:** DBS, GPi, image‐guided programming, Parkinson's disease, STN

## Abstract

**Background:**

The efficacy of deep brain stimulation (DBS) for Parkinson's disease (PD) depends on optimizing stimulation parameters for each patient, a time‐sensitive process. Image‐guided programming (IGP) offers a promising method to streamline this.

**Objective:**

The objective was to evaluate the real‐world effectiveness of an IGP tool with directional leads during the initial programming of bilateral subthalamic nucleus (STN) or globus pallidus internus (GPi) DBS in PD patients.

**Methods:**

A total of 57 PD patients (46 bilateral STN, 11 bilateral GPi) from the Vercise DBS Registry (NCT#02071134) were enrolled into the GUIDE XT substudy. Time for initial programming using IGP, Movement Disorder Society Unified Parkinson's Disease Rating Scale (MDS‐UPDRS) Part III at baseline, 6‐months, and 1‐year postactivation, Global Impression of Change from patients and clinicians, and the continued use of IGP‐suggested settings were analyzed. Stimulation field model (SFM) overlap between initial and 1‐year target volumes was also examined.

**Results:**

Motor function significantly improved at 6 months and 1 year (55% and 45%, *P* < 0.0001) compared to baseline, as assessed by MDS‐UPDRS III (Meds OFF/Stim ON). Initial programming sessions of bilateral leads using IGP (n = 56) lasted 39.4 ± 4.4 minutes (mean ± standard error [SE]), whereas it was completed in less than 30 minutes in 55% of subjects. Contact selection, polarity, and fractionalization determined at initial programing using IGP remained unchanged in 52% and 43% of subjects (n = 21) up to 6 months and 1 year, respectively. The average SFM overlap for all subjects was 92% (SE: 15%) at the 1‐year visit.

**Conclusions:**

IGP facilitates efficient initial programming sessions, providing stable settings that result in long‐term motor improvements.

Parkinson's disease (PD) is a progressive neurodegenerative disorder that affects dopaminergic transmission, leading to motor symptoms such as bradykinesia, rigidity, and resting tremor. Deep brain stimulation (DBS) targeting the basal ganglia–thalamocortical circuits, which include the subthalamic nucleus (STN) and globus pallidus internus (GPi), is an established therapy for managing motor symptoms associated with PD. The effectiveness of DBS highly depends on patient selection, lead placement, and programming of stimulation parameters. DBS programming aims to identify specific parameters (ie, amplitude, frequency, pulse width, contact selection, contact polarity, current distribution) that provide optimal motor symptom control while minimizing side effects. The gold standard for DBS programming has been the monopolar review, a process in which all contacts are systematically examined for their capacity to achieve the best possible outcome.^1^, ^2^ Although effective, this method is complex, time‐consuming for clinicians and patients, demands high levels of expertise, and often subjects patients to lengthy OFF medication testing. Additionally, the introduction of directional leads with multiple independent current control (MICC) and advanced programming features, while providing benefits for patients, has further increased the complexity of optimizing each patient's therapy by expanding the range of possible parameter combinations. Image‐guided programming (IGP) has emerged as an alternative to streamline this process while achieving good clinical outcomes.

IGP is a method‐driven technique that leverages patient‐specific neuroanatomical data of the target structures within the basal ganglia, along with the postoperative lead location and orientation. One advantage of this approach is its use of anatomical data that is readily accessible within clinical workflows, using widely available imaging modalities. Specialized computer programs, using three‐dimensional (3D) stimulation field models (SFMs), integrate this neuroanatomical information to provide a clear 3D visual representation of the position of the DBS leads, their individual contacts in relation to the target structures, and a simulation of the volume of tissue activated (VTA) based on the SFMs.

Recent studies have shown that IGP allows programmers to achieve an optimal setting in significantly less time while maintaining similar outcomes to standard‐of‐care programming.^3^, ^4^, ^5^, ^6^ Subsequent research has also indicated that IGP can help rescue patients experiencing mild side effects or suboptimal outcomes from their clinically derived existing settings by predicting more optimal stimulation parameters.^7^ In the same study, the authors demonstrated that IGP also led to significant improvements in quality of life (QoL) scores and improved patient satisfaction with the therapy. Additionally, a randomized controlled trial evaluating the acute benefits of an automated imaging‐based algorithm for STN DBS in PD was able to establish non‐inferiority when compared with standard‐of‐care treatment.^8^

Although these studies suggest that IGP is a promising approach for improving the speed of DBS programming without compromising efficacy compared to conventional clinical response testing, there is limited evidence regarding its long‐term stability and effectiveness.

In this prospective, multicenter, real‐world outcomes study, we evaluate the use of an IGP tool with directional leads during the initial programming of bilateral STN or GPi DBS in patients with PD. Here, we report the long‐term clinical outcomes, programming time, and other programming metrics, including the sustainability of the initial target volume, between patients using this IGP tool.

## Patients and Methods

### Study Design

This trial was drawn from an ongoing, prospective, multicenter, real‐world outcomes study (Vercise DBS Registry NCT#02071134) in which we analyzed PD patients implanted with a Vercise DBS system and bilateral directional 1–3–3–1 leads (Cartesia Directional Leads, Boston Scientific, Valencia, CA, USA) in either the STN or GPi. All patients provided written informed consent before they participated in the study. Preoperative planning, surgical procedures, and final confirmation of DBS lead placement were conducted by each physician's clinical judgment and were followed per standard of care. The GUIDE XT substudy of this registry further examined the effects of using IGP during initial programming of PD patients.

At the initial activation visit, subjects were programmed using Guide XT‐derived settings while programming time and therapeutic window were collected. Sites were allowed to further optimize programming and titrate amplitude while recording benefit and side effect thresholds for each setting that was tested.

After the activation visit, patients resumed the prescribed follow‐up schedule in the parent registry, including visits at 6‐month and 1‐year postactivation. The continued use of IGP was permitted at follow‐up visits throughout the duration of the study, whereas traditional monopolar review and standard‐of‐care clinical programming were also allowed at any subsequent visits to enable further exploration and optimization at clinician's discretion.

Safety was assessed throughout the study, which included capturing stimulation‐related adverse events. For safety endpoints, the rate of occurrence of all adverse events were coded using *MedDRA* (version 10.1).

### Image‐Guided Programming

Preoperative magnetic resonance imaging (MRI) and postoperative computed tomography (CT) scans were collected upon screening, prior to each patient's initial programming. Each center followed its specific imaging protocols per standard of care. At a minimum, to achieve the best segmentation results in the brain, it is recommended that at least a preoperative MR T1 (≤1 mm slice resolution, ≤1 mm slice thickness, >100 slices of the complete head) and an MR T2 or fluid‐attenuated inversion recovery (FLAIR) (≤2 mm slice resolution) sequence be collected. Postoperative CT scan recommendations include ≤1 mm in‐plane resolution without artifacts, ≤2.5 mm slice thickness, >20 slices, and high signal‐to‐noise ratio. All imaging was then processed using the Guide XT workflow (Image Fusion, Anatomical Mapping, Lead Localization, and Guide XT) provided in the *Brainlab Elements* software (Brainlab AG, Munich, Germany).

This software allows for the visualization of a VTA computed from a clinically validated SFMs within patient‐specific anatomy, along with the corresponding lead location and orientation.^9^ As a planning simulation tool, it aids in the selection of programming parameters for use with Vercise DBS systems and enables the precise shaping and steering of stimulation delivered by MICC‐capable IPGs.

**FIG. 1:**
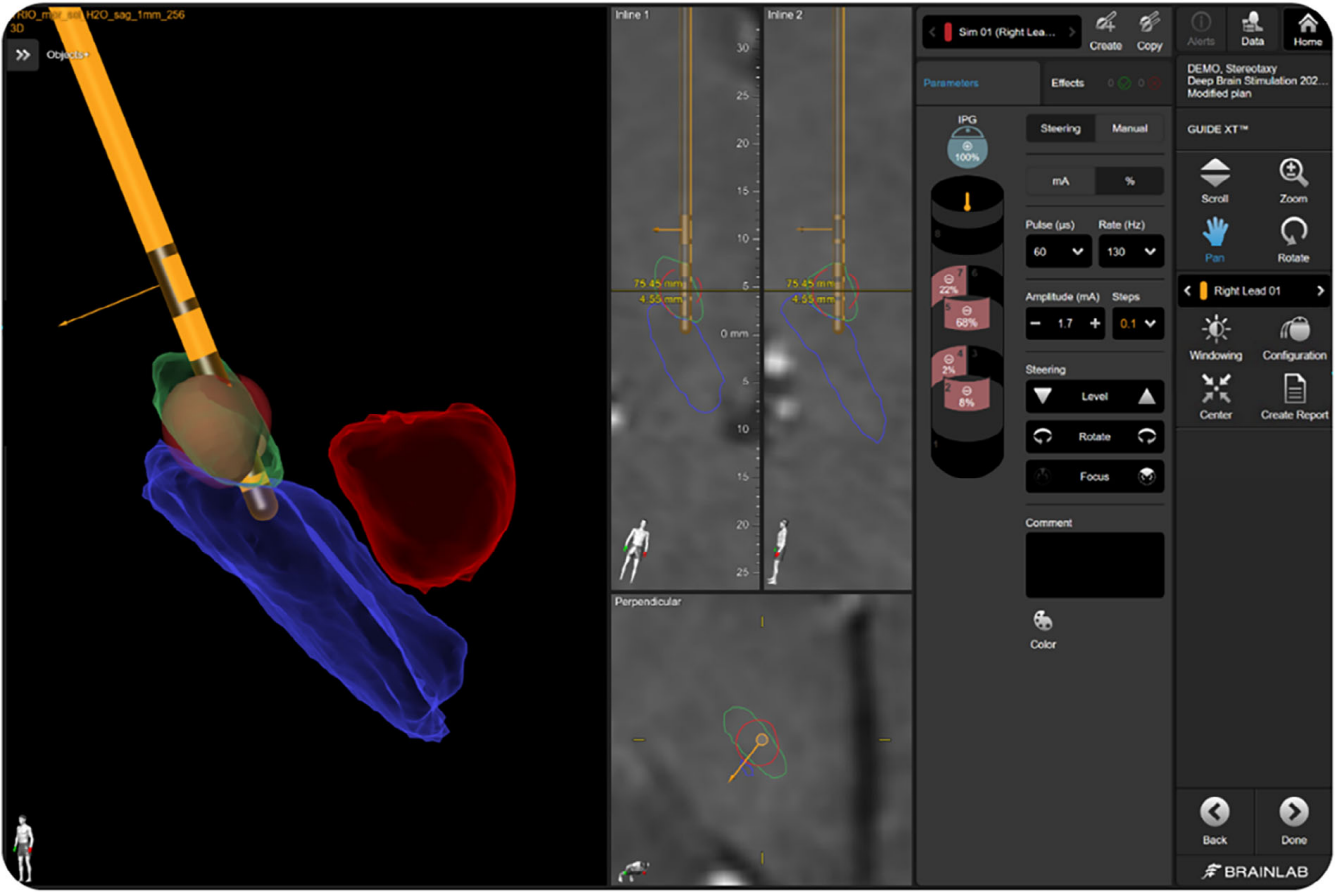
This is a screenshot taken from the *Guide XT* software in which the stimulation field model (SFM) (red volume surrounding the deep brain stimulation [DBS] lead) can be seen filling the subthalamic nucleus (STN) (green). Clinicians adjusted the available parameters seen on the right side of the screen (amplitude, pulse width, steering controls, and/or contact configuration) to fine‐tune the SFM during the initial selection of programming parameters to be used at the activation visit for each patient.

Upon completion of all image processing steps, the Guide XT module was then utilized to select the initial programming parameters. This was done by analyzing the relative lead position within the patient‐specific anatomy and selecting the contact(s) closest in proximity to the target structure. A Guide XT setting was then created using the chosen electrodes as the amplitude, and/or pulse width was adjusted while visualizing the resulting SFM (Fig. 1). Both axial and lateral steering of the SFM was performed at clinician's discretion to best fill the target region of interest while minimizing overlap with any potential side effect‐generating areas. The final settings were then documented, and this Guide XT setting was used as the starting point for programming at each patient's activation visit. Although amplitude and pulse width impact the resulting size of the SFM within the Guide XT modeling, these parameters (along with frequency) were adjusted/titrated in clinic during the activation visit when identifying benefit and side effect thresholds for each individual patient on a given program. The programming time reported below does not account for the above workflow and instead only accounts for the time spent between the clinician and patient in the clinic.

### Analysis of Programming Data

Device settings were recorded at each subject's initial activation, whereas the collection of 6‐month and 1‐year programming data was optional per the registry protocol. Programming time was documented at the initial activation, and other parameters such as electrode configuration, amplitude, frequency, and pulse width were extracted from the programming reports to be compared across visits. Programming types were classified as directional versus nondirectional, bipolar versus monopolar, and whether MICC was being used. As a result, all programs were labeled using a combination of the above classifications that are not mutually exclusive and describe different features of the programming being implemented (ie, a single program can be defined as directional, monopolar, and non‐MICC).

Directional programming is defined here as any setting involving a level with segmented electrodes (levels 2 and 3 for DB‐2202) in which the current assigned was not spread equally among all three directional contacts, opposed to a ring mode configuration. Bipolar programming was any configuration in which both cathodic and anodic currents were assigned to contacts on the lead (a percentage of current was allowed to remain on the IPG). Finally, the use of MICC included any instance where current is placed on more than one contact and is not equally distributed (ie, using two active contacts in which the percentage of current isn't split with 50% on each, such as 25% and 75%, 40% and 60%, etc.).

These metrics were then compared between visits to determine how much each variable changed over the first year of programming. A nonparametric McNemar's test was used to analyze the paired nominal data for the programming types listed above.

### SFM Overlap Analysis

SFMs were generated to assess the sustainability of the initial target volume compared to 1‐year postactivation. This analysis was carried out using Brainlab Elements, with the Guide XT module used to generate SFMs and the Object Manipulation module to calculate their resulting overlap. The sustainability of the initial target volume using IGP was then determined by calculating the percentage overlap of the SFMs between activation (ACT) and the 1‐year (1YR) visit using Equation (1). The results were averaged and grouped by target structure to provide further characterization.

(1) $$\begin{array}{l} \%\text{Overlap}=\frac{A\cap B}{\min\;\left(A,B\right)}\\ \text{Where}\;A={\text{Volume}}_{ACT}\;\text{and}\;B={\text{Volume}}_{1\mathrm{YR}} \end{array}$$

### Outcome Measures

Study endpoints included the time to reach effective stimulation settings using IGP at the activation visit, Movement Disorder Society Unified Parkinson's Disease Rating Scale (MDS‐UPDRS) Part III, Clinical Global Impression of Change (CGI‐C) as assessed by the clinician, Clinical Global Impression of Change—Subject (CGI‐C: Sub), and the use of IGP‐suggested settings, all taken from baseline to 6 months and 1‐year postactivation. A nonparametric Wilcoxon test was used to evaluate clinical outcomes, whereas an independent samples *t* test was used when comparing initial programming times between targets (using *SAS* software).

## Results

### Patient Demographics

Fifty‐seven PD patients (mean age: 62.9 ± 8.8 years) who underwent DBS implantation were included in the study; 22 subjects had their leads implanted under awake conditions and 35 under asleep conditions. Of these patients, 46 received bilateral STN DBS and 11 received bilateral GPi DBS. Forty‐four subjects were male and 13 were female, with a mean disease duration of 10.1 ± 5.6 years for all subjects.

### Initial Programming Time

**FIG. 2:**
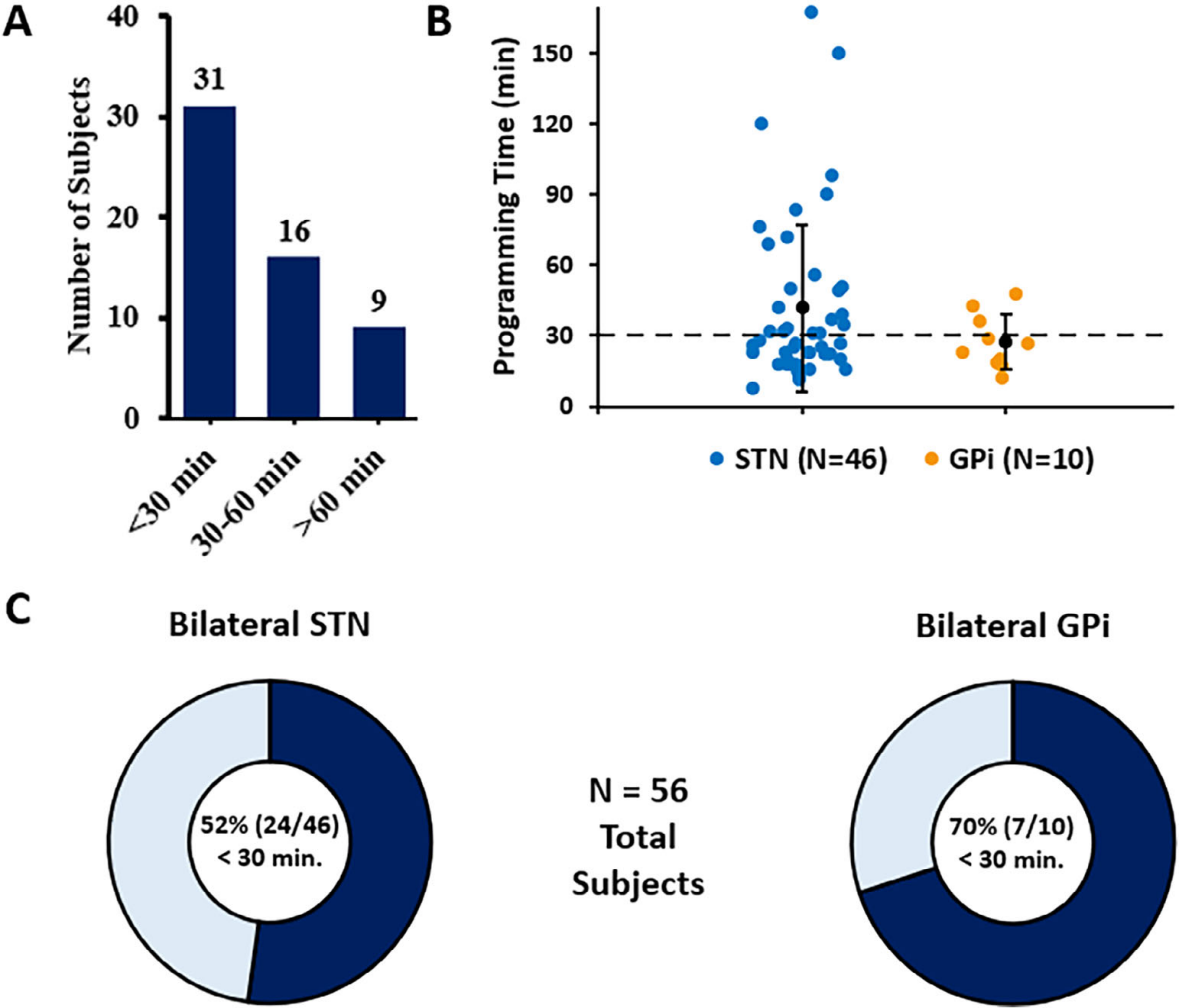
This figure illustrates the time spent programming both leads during the initial activation visit using image‐guided programming (IGP). The number of subjects with programming times < 30‐, 30–60‐, and >60 minutes is shown in (**A**) along with a scatter plot of all programming times by target (average ± standard deviation per target also shown) in (**B**). (**C**) The percentage of subjects programmed in <30 minutes per target structure is shown.

Initial programming sessions (postimplant), where IGP was used to create the settings for bilateral directional leads (N = 56, with one subject left out due to missing programming times), took an average of 39.4 ± 4.4 minutes (mean ± stand error [SE]). From this cohort, 55% (31/56) completed initial programming in less than 30 minutes. When programming time was analyzed by target, STN patients had an average of 42.0 ± 5.2 minutes, whereas GPi patients demonstrated slightly faster programming sessions with an average time of only 27.4 ± 3.7 minutes (*P* = 0.027, independent samples *t* test). Additional results are shown in Figure 2, along with a separation by target structure.

### Long‐Term Stability and Programming Types with IGP

**FIG. 3:**
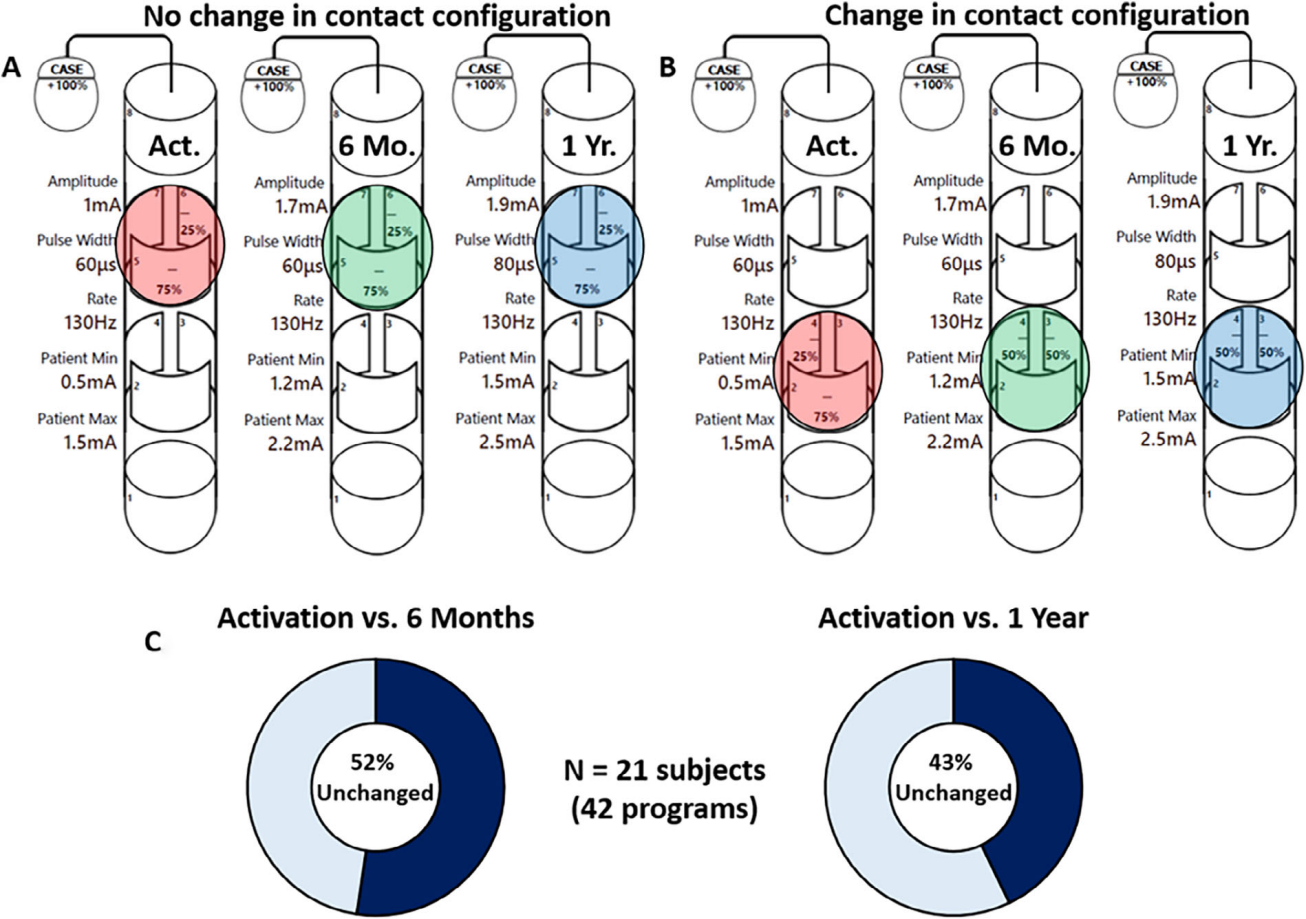
This figure shows examples of programming settings taken at activation, 6 months, and 1 year. (**A**) An example of one subject's programming for the same lead at each time point, showing that no changes occur in the chosen contact configuration (highlighted contacts: red—activation, green—6 months, and blue—1 year). Another example in which the contact configuration does change can be seen in (**B**). The percentage of all programs that did not change from the activation visit to 6 months and 1 year can also be seen in (**C**). A change in this instance was defined as one made to the contact(s) used for programming and the % of cathode/anode, whereas changes to other settings, including amplitude, pulse width, or rate, were not included in the above analysis.

**FIG. 4:**
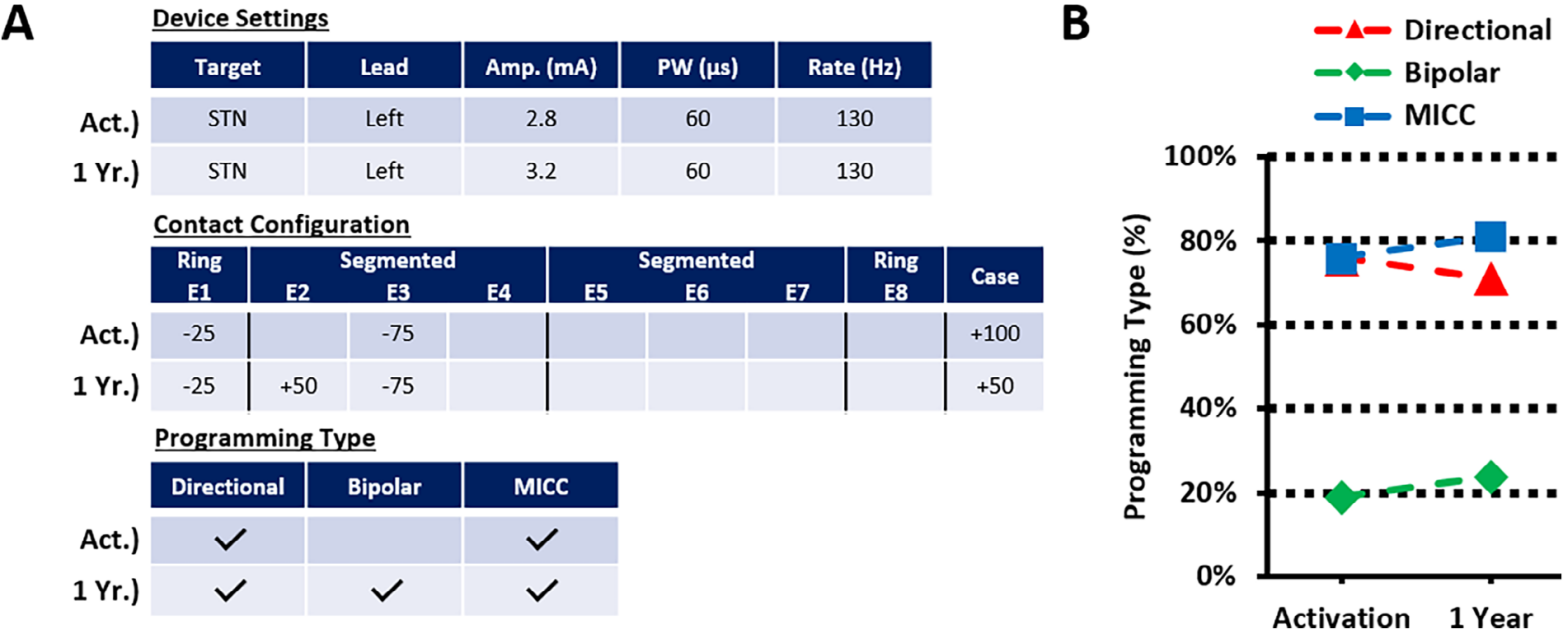
An example of the device settings, contact configuration, and programming type extracted from one subject's programming report (left lead only in this example) at activation and 1 year are shown in (**A**), and a plot of programming type for all subjects (N = 21) is plotted in (**B**). No significant differences (*P* < 0.05) were found between activation and 1 year for any of the programming types listed (directional: *P* = 0.563, bipolar: *P* = 0.654, and multiple independent current control [MICC]: *P* = 0.563) using McNemar's test.

In this cohort, 21 bilaterally implanted subjects (42 leads = 42 programs) had programming reports submitted at the 1‐year follow‐up visit. At 6 months and 1 year, 52% (22 of 42) and 43% (18 of 42) of the active contacts and their assigned current distribution remained unchanged from the initial activation visit while using IGP. This indicates that the initial choice of contact(s) and the cathodic/anodic distribution of current remained the same for these programs at each time point. These findings, along with an example case, are illustrated in Figure 3 for reference. Additional programming changes made between activation and 1 year are shown in Table 1, with a separation by target. We also examined the types of programming used using IGP for all 21 subjects with available data. When categorizing the programming types at initial activation and 1‐year follow‐up, 76% (16 of 21) and 71% (15 of 21) of subjects were using a directional program (*P* = 0.563), 19% (4 of 21) and 24% (5 of 21) of subjects included a bipolar setting (*P* = 0.654), and 76% (16 of 21) and 81% (17 of 21) of subjects had a program created using MICC (*P* = 0.563), respectively. An example of this is shown in Figure 4, along with the activation and 1‐year averages for each programming type. An additional breakdown of programming types comparing each target can also be found in Table 1. These findings demonstrate the ability of IGP to selectively target an optimal location, both at initial programming and during long‐term follow‐up, with the assistance of the provided features.

**TABLE 1:** Clinical demographics, outcomes, and programming details by deep brain stimulation (DBS) target

	STN (N = 46)	GPi (N = 11)
Age (y)	64.7 ± 7.8	55.6 ± 9.6
Disease duration (y)	9.9 ± 4.7	10.7 ± 8.6
Gender, (n/N) male	36/46	8/11
Programming time at activation (min) (mean ± SE)	42.0 ± 5.2	27.4 ± 3.7
MDS‐UPDRS III Meds Off (mean ± SD, n)		
Baseline visit	48.5 ± 14.0, 44	46.3 ± 10.6, 11
6‐month visit	21.2 ± 11.4, 37	21.3 ± 10.8, 8
Change between 6 month and baseline, % improvement	55.5%	53.2%
1‐year visit	24.4 ± 12.8, 29	26.8 ± 8.4, 8
Change between 1 year & baseline, % improvement	46.4%	42.1%

Programming type (n/N)	STN (N = 15)	GPi (N = 6)
Activation—directional^a^	73% (11/15)	83% (5/6)
Bipolar^b^	0% (0/15)	67% (4/6)
MICC^c^	73% (11/15)	83% (5/6)
1 year—directional^a^	60% (9/15)	100% (6/6)
Bipolar^b^	20% (3/15)	33% (2/6)
MICC^c^	73% (11/15)	100% (6/6)
Programming changes between activation and 1 year (n/N)		
Amplitude	100% (15/15)	100% (6/6)
Pulse width	40% (6/15)	83% (5/6)
Rate	40% (6/15)	0% (0/6)

^a^ Directional programming was defined as any setting involving a level with segmented electrodes (levels 2 and 3, DB‐2202) in which the current was not spread equally among all three directional contacts.

^b^ Bipolar programming was defined as any configuration in which both cathodic (−) and anodic (+) currents were assigned to contacts on the lead.

^c^ MICC included any instance where current is placed on more than one contact and is not equally distributed.

Abbreviations: STN, subthalamic nucleus; GPi, globus pallidus internus; MDS‐UPDRS III, Movement Disorder Society Unified Parkinson's Disease Rating Scale Part III, MICC, multiple independent current control.

### SFM Overlap Comparison

SFMs correlating with each subject's activation and 1‐year programming were generated and compared to determine how much of the initial target volume was still being used after 1 year of additional programming and optimization.

Out of the 42 total programs (21 subjects), 36 programs resulted in a measurable SFM volume, whereas the remaining 6 programs were excluded from this analysis. An SFM volume of zero can occur within the *Guide XT* software if the simulated volume is less than 0.01 cm^3^ (calculation based on the stimulation parameters), which is smaller than the software's output resolution. This happens when the chosen settings produce an output below the activation threshold specified in the modeling parameters (eg, when low amplitudes are applied and/or when the current is distributed across multiple contacts).

**FIG. 5:**
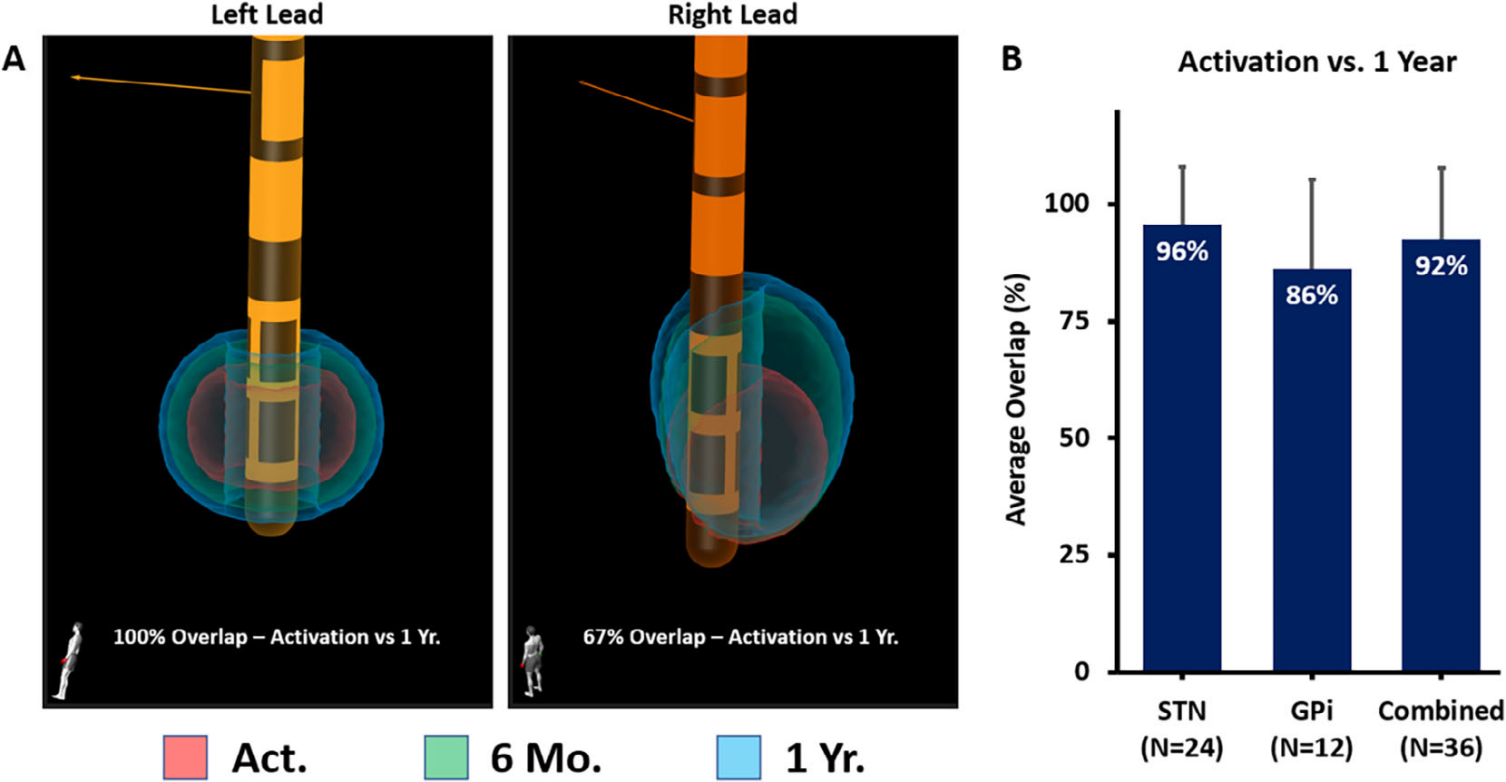
Screenshots taken from a GUIDE XT subject, showing each lead's representative stimulation field models (SFMs) at activation (red), 6‐month (green), and 1‐year (blue) visits, along with the resulting overlap between activation and 1 year, are shown in (**A**). The average overlap for all programs when comparing activation and 1 year is shown in (**B**), along with the same results when separated by target (mean ± standard deviation).

Among the 36 programs, 24 were STN leads and 12 were GPi, all of which were included in the overlap comparison. The average overlap across all subjects (36 total leads) was 92% ± 15.2%. When analyzed by target, the SFM overlap for STN was 96% ± 12.2% and for GPi was 86% ± 19.0% (Fig. 5).

### Sustained Clinical Outcomes

**FIG. 6:**
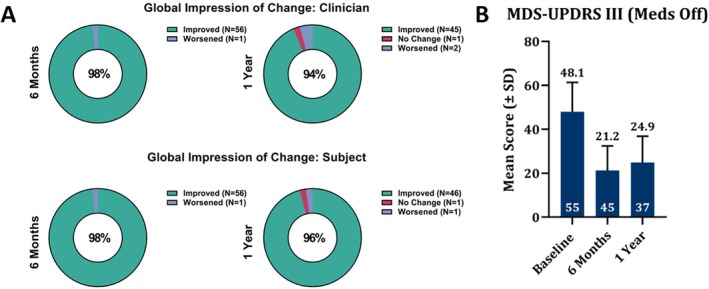
The plots above in (**A**) show the Global Impression of Change by Clinician and Subject at 6 months and 1 year, respectively, and the sustained improvement in motor function out to 1 year is shown in (**B**) (Wilcoxon's nonparametric test, *P* < 0.0001).

Significant improvements in motor function compared to baseline were observed at 6 months and sustained through 1 year (*P* < 0.0001, Fig. 6), as measured by MDS‐UPDRS III (Meds OFF/Stim ON). Results showed a 55% improvement at 6 months and a 45% improvement at 1 year. Additionally, over 98% of both subjects and clinicians reported an improvement in PD symptoms with DBS therapy at 6 months, which was sustained up to 1 year; see Table 1 for additional clinical outcome breakdown by target. Additionally, of the 21 subjects with 1‐year follow‐up, 6 subjects had unchanged current distributions from the initial IGP setting at activation through 1 year that resulted in similar improvement in motor function observed at 6 months and 1 year, respectively (see Supplementary Appendix).

## Discussion

The objective of the GUIDE XT substudy was to assess the use of Guide XT as a planning tool during initial programming, the stability of the proposed settings, and the long‐term (up to 1 year) motor outcomes. Specifically, we aimed to explore how visualizing SFMs within patient‐specific anatomy could assist in programming STN and GPi DBS.

This study provided valuable insights into IGP, answering key questions regarding (1) long‐term efficacy data from a larger, multicenter, real‐world cohort, (2) differences in programming between approved anatomical targets for PD (eg, STN vs. GPi), and (3) the sustainability of IGP‐suggested settings.

Results from this prospective, open‐label, multicenter study showed that initial programming sessions for bilateral leads took an average of 39.4 minutes (n = 56), with 55% of subjects completing programming in under 30 minutes. When comparing targets, 52% of bilateral STN and 70% of bilateral GPi programming were completed in under 30 minutes, with GPi patients reporting lower initial programming times on average (42.0 ± 5.2 minutes vs. 27.4 ± 3.7 minutes, *P* = 0.027). Possible reasons for this may be supported by reports that the larger GPi target is thought by some to be easier to program relative to the smaller more compact STN, allowing more latitude for lead placement and a lower likelihood of spreading current into undesirable adjacent regions and pathways.^10^ These findings suggest that initial DBS programming using a patient‐specific anatomical target may be faster than prior standard‐of‐care methods that rely on monopolar testing (90–240 minutes).^5^, ^11^

Without patient‐specific anatomy, an MICC‐capable IPG with directional DBS leads offers a wide range of potential programming parameters but may require multiple programming sessions before a stable program is reached. This can result in programmer and patient fatigue, potentially resulting in suboptimal outcomes. In this study, we showed that programs derived using IGP at initial activation remained unchanged for many subjects (52% and 43% of programs were unchanged at 6 months and 1 year, respectively). In those patients where there was a change, the resulting initial target volume (SFM) remained relatively stable over the course of a year. The average overlap between SFMs at these time points was 92% for the overall population, with 96% overlap in the STN and 86% in GPi targets. Although IGP was only required at activation, clinicians had the option to either revert to their standard‐of‐care approach or continue with IGP based on the patient's needs at follow‐up visits. Even though we did not capture which programming methods were chosen at subsequent visits postactivation, the SFM analysis indicated minimal fluctuation from the initial target volume derived using IGP.

We also analyzed how IGP facilitated the use of directional programming, bipolar settings, and MICC, observing that at activation, 76% of subjects were using a directional program, 19% included bipolar settings, and 76% were utilizing MICC. The use of MICC is exclusive to this system under study and through the use of fractionated anode and cathode allows highly refined best fit for model to patient‐specific anatomy. These features were consistently maintained at the 1‐year follow‐up, demonstrating that IGP enabled clinicians to accurately target an optimal location during the initial programming session, with results lasting at least 1 year. This suggests that combining IGP with MICC at activation allows for precisely defined targets, requiring only minor adjustments (eg, small changes in amplitude or contact fractionalization) during follow‐up visits. Additionally, recent studies have shown that IGP can facilitate reprogramming in existing patients with poor clinical outcomes^7^ and even reduce hospital stays postprogramming.^12^

This study observed shorter, more efficient initial programming sessions utilizing IGP‐suggested settings and demonstrated sustained, clinically significant motor improvements. At 6 months, a mean 55% improvement in MDS‐UPDRS III (Meds OFF, Stim ON) was observed, which was maintained out to 1 year (45% improvement). These results suggest that IGP is noninferior to standard of care,^13^ although a direct comparison was not tested in this study. Patient and clinician satisfaction with DBS therapy remained high throughout the evaluation period, with approximately 98% of subjects and clinicians reporting an improvement in motor symptoms at 6 months, which was maintained at 1 year.

### Safety Reporting

A safety analysis showed no adverse events related to the activation of DBS using IGP within the GUIDE XT substudy, and only one reported an adverse event between initial programming and 1‐year follow‐up that was considered possibly related to stimulation, which resolved.

### Limitations

A major limitation of this study is the absence of a standard‐of‐care comparison group. However, prior studies^4^, ^5^, ^11^ have shown that there is no worsening in clinical outcomes between IGP and traditional programming.

Additionally, our analysis was limited to using percentage overlap when comparing SFM volumes, instead of a more commonly reported similarity measure such as the DICE coefficient. During our analysis of programming changes from activation to 1 year, we found an average increase of 53% in SFM volume across all subjects (Table 2), primarily due to gradual increases in amplitude or pulse width over time. This increase in SFM volume, potentially due to disease progression or medication adjustments, led to a decrease in the DICE coefficient, even if no changes were made to the electrode configuration or current distribution. Therefore, we used percent SFM overlap as a better indicator of initial target volume stability.

**TABLE 2:** Stimulation field model (SFM) volume from activation to 1 year by deep brain stimulation (DBS) target

	Activation volume (cm^3^)	1‐year volume (cm^3^)	Average increase
STN (N = 24^a^ SFMs)	0.041 ± 0.03	0.063 ± 0.03	54%
GPi (N = 12 SFMs)	0.122 ± 0.04	0.187 ± 0.03	53%

^a^ Six SFMs left out of STN overlap analysis due to simulated volume being less than the software's output resolution of 0.01 cm^3^.

Abbreviations: STN, subthalamic nucleus; GPi, globus pallidus internus.

When comparing programming changes between activation, 6 months, and 1 year, we defined a change as any modification in the contact configuration (eg, changes in active contacts or cathode/anode fractionalization) and excluded adjustments to parameters such as pulse width, rate, or amplitude. The reason we chose this definition is that IGP is meant to provide an accurate target location, whereas the other parameters are adjusted in clinic based on patient feedback. When analyzing the current distribution in the 21 subjects with 1 year programming data, we observed that in both subjects with unchanged (6 subjects) and changed (15 subjects) current distribution, there was an improvement in motor outcomes with DBS. However, although the improvements were similar at 6 months and 1 year, the baseline UPDRS III scores between each group appear clinically different. It is an interesting observation, but given the limited N, it is difficult to draw a meaningful conclusion, and further investigation is required.

Another limitation is that we did not evaluate the experience level of the programmers prior to IGP, so it remains unknown if this could have improved the ability of an inexperienced programmer to similarly arrive at clinically durable settings more efficiently. Tools such as IGP could potentially be used to help support less‐experienced clinicians by providing a starting point for programming and narrowing the parameter space down to a specific target area; however, additional studies are needed to explore this further.

Additionally, the *Guide XT* software was used on a stand‐alone machine throughout the study, requiring the manual transfer of any suggested program settings to the clinician programmer. However, with recent updates, this process can now be completed in a more streamlined workflow using the *Vercise Neural Navigator 5* Software (VN5) without the need to manually transfer settings.

In conclusion, as DBS technology advances and the access to DBS expands, our study found that IGP was an effective tool for obtaining robust clinical benefit. Shorter programming times and more stable initial programming over time will help improve the practicality and applicability of this technology.

## Author Roles

(1) Research project: (A) Conception; (B) Organization; (C) Execution; (2) Data Collection and analysis: (A) Design; (B) Execution; (C) Review and critique; (3) Manuscript preparation: (A) Writing; (B) Final review and critique.

J.L.A.: 1A, 1C, 2A, 2B, 2C, 3A, 3B; TZ: 1B, 1C, 2B, 2C, 3B

M.S.O.: 1B, 1C, 2B, 2C, 3A, 3B

A.R.Z.: 1B, 1C, 2B, 2C, 3B

O.E.V.: 1B, 1C, 2B, 2C, 3B

L.V.M.: 1B, 1C, 2B, 2C, 3A, 3B

C.C.L.: 1B, 1C, 2B, 2C, 3B

R.R.: 1B, 1C, 2B, 2C, 3B

J.D.: 1B, 1C, 2B, 2C, 3B

Y.B.B.: 1B, 1C, 2B, 2C, 3B

J.D.C.: 1B, 1C, 2B, 2C, 3B

K.D.F.: 1B, 1C, 2B, 2C, 3B

S.B.S.: 1B, 1C, 2B, 2C, 3B

A.M.P.: 1B, 1C, 2B, 2C, 3B

J.R.J: 1B, 1C, 2B, 2C, 3B

D.B.W.: 1B, 1C, 2B, 2C, 3B

J.P.: 1B, 1C, 2B, 2C, 3B

A.H.: 1A, 1B, 1C, 3A, 3B

R.S.S.: 1A, 1B, 1C, 2A, 2C, 3A, 3B

B.R.: 1A, 1B, 1C, 2A, 2C, 3A, 3B

E.G.: 1A, 1B, 1C, 2A, 2C, 3B

## Disclosures

**Ethical Compliance Statement**: This study was approved by each institution's local Institutional Review Board (IRB). All patients provided their written consent for the research, documentation, and publication. We confirm that we have read the journal's position on issues involved in ethical publication and affirm that this work is consistent with those guidelines.

**Funding Sources and Conflicts of Interest:** Boston Scientific provided funding for the study and contributed to the study design, monitoring, and data management. The sponsor had no role in data collection for the study. The Clinical Research Team (A.H., R.S.S., B.R., E.G.) at Boston Scientific supported the analysis and provided administrative and technical support as needed during the study. The authors declare that there are no conflicts of interest relevant to this work.

**Financial Disclosures for the Previous 12 Months**: J.L.A. reports consulting and speaker honoraria from Boston Scientific for the submitted work; T.Z. reports no disclosures; M.S.O. has an unpaid consulting agreement for data analysis and interpretation with Boston Scientific; A.R.Z. reports no disclosures; O.E.V. reports no disclosures; L.V.M. reports consulting fees for Abbott; C.C.L. reports no disclosures; R.R. reports consulting fees for Encora Therapeutics; J.D. reports no disclosures; Y.B.B. reports receiving an education grant from Medtronic, consulting fees from Medtronic and PMT Corporation, speaker honoraria from Boston Scientific and Abbott, and travel reimbursement from Medtronic and Abbott; J.D.C. reports no disclosures; K.D.F. reports no disclosures; S.B.S. reports receiving fellowship training grants from Abbott, Boston Scientific, Medtronic, and royalties from Globus Medical; A.M.P. reports no disclosures; J.R.J. reports receiving an ISR‐grant support from Boston Scientific and speaker honoraria from Abbott and Boston Scientific; D.B.W. reports no disclosures; J.P. reports receiving grants from Abbott, Boston Scientific, Focused Ultrasound Foundation, Medtronic, and National Institute of Health (R01EB030324, 5U54EB033650, R18EB036591)—sitting on the Data Safety and Monitoring Board and holding stock options for AIM Medical Robotics. A.H., R.S.S., B.R. and E.G. are paid employees of Boston Scientific.

## Supporting information


**Data S1.** Supporting information.

## Data Availability

The data that support the findings of this study are available on request from the corresponding author. The data are not publicly available due to privacy or ethical restrictions.
