# Supplementary material for: Sustained Therapeutic Benefits Using Image‐Guided Programming at Activation of Deep Brain Stimulation for Parkinson's Disease
Source: Mov Disord Clin Pract. 2025 Jun 5;12(11):1821–30. doi: 10.1002/mdc3.70154 (PMC12625106; doi:10.1002/mdc3.70154)
Supplement: Supplementary file 1 — Data S1. Supporting information. [file MDC3-12-1821-s001.docx]

**Supplementary Appendix**

This appendix formed part of the original submission and has been peer reviewed. We post it as supplied by the authors

**Supplement to:** Aldred J.L., et al. Sustained Therapeutic Benefits Using Image-Guided Programming at Activation of Deep Brain Stimulation for Parkinson's Disease

### **Table A1.** Clinical Outcomes by current distribution

|  | Unchanged Current Distributions  (N=6) | Changed Current Distributions (N=15) |
| --- | --- | --- |
| **MDS-UPDRS III Meds Off (mean** ± **SD, n)** |  |  |
| Baseline Visit | 39.2 ±13.7, 6 | 50.6 ± 14.8, 14 |
| 6 Month Visit | 24.3 ± 10.0, 6 | 27.6 ± 14.4, 11 |
| 1 Year Visit | 27.0 ± 5.0, 6 | 24.6 ± 7.9, 11 |
